# A Study on REM Sleep Homeostasis in the Day-Active Tree Shrew (*Tupaia belangeri*): Cold-Induced Suppression of REM Sleep Is Not Followed by a Rebound

**DOI:** 10.3390/biology12040614

**Published:** 2023-04-18

**Authors:** Sjoerd J. van Hasselt, Luisa Epifani, Danique Zantinge, Kornelija Vitkute, Martien J. H. Kas, Giancarlo Allocca, Peter Meerlo

**Affiliations:** 1 Groningen Institute for Evolutionary Life Sciences, University of Groningen, 9747 AG Groningen, The Netherlands; 2 School of Biomedical Sciences, University of Melbourne, Parkville, VIC 3010, Australia; 3 Somnivore Pty. Ltd., Bacchus Marsh, VIC 3340, Australia

**Keywords:** sleep homeostasis, REM sleep, sleep deprivation, body temperature, brain temperature, cold exposure, tree shrew, diurnal, EEG

## Abstract

**Simple Summary:**

The function and regulation of rapid-eye-movement (REM) sleep is a topic of ongoing debate. It is often assumed that REM sleep is a homeostatically regulated process and that a need for REM sleep builds up, either during prior wakefulness or during preceding slow wave sleep. In the current study, we tested this hypothesis in the day-active tree shrew (*Tupaia belangeri*), a small mammal closely related to primates. We exposed the animals to a low temperature of 4 °C during their main sleep phase, a procedure that is known to suppress REM sleep. Cold exposure caused a significant drop in brain temperature and body temperature and also resulted in a strong and selective suppression of REM sleep. However, contrary to our expectation, the loss of REM sleep was not recovered during the subsequent day and night. These findings in a day-active mammal confirm that the expression of REM sleep is highly sensitive to environmental temperature but do not support the view that REM sleep is homeostatically regulated in this species.

**Abstract:**

The function and regulation of rapid-eye-movement (REM) sleep is a topic of ongoing debate. It is often assumed that REM sleep is a homeostatically regulated process and that a need for REM sleep builds up, either during prior wakefulness or during preceding slow wave sleep. In the current study, we tested this hypothesis in six diurnal tree shrews (*Tupaia belangeri*), small mammals closely related to primates. All animals were individually housed and kept under a 12:12 light-dark cycle with an ambient temperature of 24 °C. We recorded sleep and temperature in the tree shrews for 3 consecutive 24 h days. During the second night, we exposed the animals to a low ambient temperature of 4 °C, a procedure that is known to suppress REM sleep. Cold exposure caused a significant drop in brain temperature and body temperature and also resulted in a strong and selective suppression of REM sleep by 64.9%. However, contrary to our expectation, the loss of REM sleep was not recovered during the subsequent day and night. These findings in a diurnal mammal confirm that the expression of REM sleep is highly sensitive to environmental temperature but do not support the view that REM sleep is homeostatically regulated in this species.

## 1. Introduction

Sleep is a quiescent state characterized by reduced responsivity to environmental stimuli [1,2,3]. In mammals and birds, sleep consists of two different and alternating stages called non-rapid eye movement (NREM) sleep and rapid eye movement (REM) sleep. These two forms of sleep can be distinguished from each other and from wakefulness on the basis of electrophysiological recordings of brain activity (electroencephalogram, EEG) and muscle activity (electromyogram, EMG). Both NREM and REM sleep are assumed to serve important functions, but what these functions are is still a topic of ongoing research and debate [4,5,6,7,8].

Several of the popular theories propose that sleep is a state during which physiological or neuronal maintenance processes take place to recover from prior wakefulness. This idea is supported by numerous studies indicating that a drive for sleep builds up during the time awake and further increases with sleep deprivation, suggesting that sleep is a homeostatically regulated process [4,9].

The homeostatic regulation of NREM sleep in mammals is well established [9]. After sleep deprivation, there is a clear rebound in NREM sleep time and intensity, with the latter being reflected in a dose-dependent increase in EEG slow-wave-activity (SWA) in the 1–4 Hz frequency range [10,11,12].

In contrast, the regulation of REM sleep is still a topic of debate, and sleep deprivation studies have produced inconsistent findings with regard to REM sleep rebounds [9]. Some studies showed that REM sleep increases after sleep deprivation [13,14,15], while in others, rebounds were inconsistent, small or absent [16,17,18]. Some papers also reported changes in REM sleep EEG characteristics after sleep deprivation, particularly in the 7–10 Hz theta range, suggesting that the quality and perhaps intensity of REM sleep may change as well in response to sleep deprivation [15,17]. However, these changes were subtle and, in some cases, also occurred in the waking state [15]. It is also unclear if these changes depend on the duration of sleep deprivation and indeed reflect REM sleep homeostasis. In fact, it is still generally believed that the recovery of lost REM sleep mostly occurs through an increase in REM sleep time but may only occur after longer sleep deprivation [9].

The traditional view on sleep homeostasis is that the pressure for both NREM and REM sleep accumulates during the waking phase [19]. According to this view, extended wakefulness, i.e., sleep deprivation, would result in a compensatory rebound of both NREM and REM sleep in the recovery phase thereafter. Benington and Heller (1994) proposed an alternative model according to which REM sleep is functionally related to prior NREM sleep rather than prior wakefulness [20]. This model suggests that the pressure and need for REM sleep does not accumulate during waking but rather increases during NREM sleep. This model could perhaps explain some of the inconsistencies of REM sleep regulation reported in the literature. When subjects are exposed to total sleep deprivation, there would not be a build-up of REM sleep pressure and hence no need for a rebound. On the other hand, when subjects experience sleep fragmentation or other forms of sleep disturbance that allow for (some) NREM sleep, they would gradually build up REM sleep pressure. Similarly, selective REM sleep deprivation, leaving NREM sleep largely unaffected, would lead to a rapid increase in the pressure and need for REM sleep.

In the current study, we investigated REM sleep regulation in the tree shrew (*Tupaia belangeri*), a day-active mammal originating from south-eastern Asia [21]. The tree shrew belongs to the order of Scandentia, making it closely related to primates and flying lemurs [22,23]. This animal may be a useful model species for sleep research, as their sleep structure is more similar to that of humans than to that of the commonly used nocturnal laboratory rodents [18,24]. An earlier study on sleep homeostasis in the tree shrew showed that sleep deprivation for 6 or 12 h resulted in a partial NREM sleep rebound but no recovery of the REM sleep that was lost [18]. This finding does not support the view that a homeostatic pressure for REM sleep builds up during wakefulness, but it could be in line with the view that REM sleep pressure builds up during preceding NREM sleep. We therefore aimed to test whether, in the tree shrew, the selective suppression of REM sleep, leaving NREM sleep intact, would be followed by a rebound of REM sleep thereafter.

We used cold exposure as an approach for selective REM sleep deprivation, a procedure that has been well established and validated in cats and rats [25,26,27,28]. We exposed tree shrews equipped with EEG loggers to a low temperature of 4 °C during the nighttime, their main sleep phase, and assessed whether this would result in REM sleep suppression and a subsequent REM sleep rebound.

## 2. Materials and Methods

### 2.1. Animals and Housing

For this study, we used six tree shrews (*Tupaia belangeri*) (one female and five males). Throughout the experiments, the animals were individually housed in steel cages (w × d × h = 50 × 80 × 125 cm) that were enriched with a wooden nest box at the base of the cage (18 × 15 × 15 cm) and three wooden branches that were attached at various heights. The cages were placed in a climate cell where the ambient temperature could be controlled. The baseline room temperature was set at 24 °C, and the animals were kept under a 12 h:12 h light-dark cycle (lights on: 8 a.m.). Food and water were provided ad libitum. All procedures were approved by the national central authority for scientific procedures on animals (CCD) and the institutional animal welfare body (IVD, University of Groningen, The Netherlands), approval code: AVD10500202115448.

### 2.2. Surgery

All animals underwent surgery for the implantation of EEG/EMG electrodes to assess sleep–wake patterns and a temperature pill to measure the core body temperature. At the start of the surgery, isoflurane was used to induce general anesthesia (induction: 5%; maintenance: 0.5–2.5%) using an inhalation mask. The animals were placed on a heating mat to stabilize the core body temperature around 38 °C. Meloxicam (5 mg/mL) was injected subcutaneously to serve as analgesics. To maintain anesthesia, a subcutaneous injection of ketamine (100 mg/mL) and medetomidine (1 mg/mL) was administered. Isoflurane was then lowered to approximately 0.1% and was oxygen-enriched. For implanting the temperature pill (Anipill; Biocap, Caen, France), the abdomen was shaved, and lidocaine (2 mg/mL) was applied on the exposed area that served as additional local analgesics. A small incision was carefully made through the skin and the muscle layer underneath. After inserting the pill in the abdominal cavity, the muscle layer and the skin were closed using surgical sutures. Recorded temperatures were stored locally on the pill and were wirelessly transmitted to a monitor that was near the animals (Anipill; Biocap, Caen, France).

Subsequently, surgery continued with the implantation of EEG/EMG electrodes. The skull was carefully exposed by shaving the head of the animals, and a midline incision of approximately 2 cm was made after applying lidocaine (2 mg/mL). Four small holes were drilled (0.5 mm in diameter) in the skull to insert the electrodes to the level of the dura mater. Two frontal EEG electrodes were placed 4 mm lateral to the midline of each hemisphere. Additionally, one reference and one ground electrode were placed caudally near the cerebellum. For two animals, an additional hole was drilled (2 mm lateral and caudal to the frontal left electrode) for the placement of a thermistor to measure brain temperature (B57540, TDK electronics; Munich, Germany). In all animals, an additional 0.6 mm screw was placed over the right hemisphere, which was used as an anchor point for the implant. Furthermore, a flexible wire was subcutaneously inserted into the neck muscle for EMG recordings. All the electrodes were connected to a seven-pin plug (BKL electronic 10120302, Lüdenscheid, Germany), which was then fixed using dental cement (Paladur; Heraeus Kulzer, Hanau, Germany). A light-weight protective plug was attached to the head implant to protect the pins (BKL electronic 10120602, Lüdenscheid, Germany). At the end of the surgery, the animals received a subcutaneous injection of atipamezole (5 mg/mL) to counteract the effect of medetomidine. The animals were given two weeks to recover from surgery, during which they were closely monitored.

### 2.3. Experimental Design

**Figure 1:**
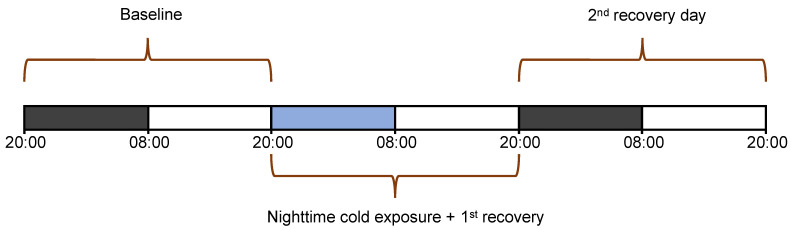
Experimental design consisting of 72 h recordings. The animals were individually housed under 24 °C and a 12:12 h LD cycle, where the black and white bars denote the dark and light phase, respectively. The light-blue bar denotes the night during which the ambient temperature was lowered from 24 to 4 °C. During the remaining 36 h, the ambient temperature was restored to baseline levels (24 °C).

Prior to the start of the experiment, around noon, miniature data loggers (Neurologger 2A; Evolocus; Tarrytown, NY, USA) containing an onboard three-axis accelerometer (LIS302DLH; STMicro-electronics Geneva, Switzerland) were connected to the implants of the animals. EEG, EMG and accelerometery data were recorded and stored on a memory chip in the logger. The core body temperature was measured and stored by the temperature pill throughout the experiment. Each animal was subjected to a 3-day recording session. We recorded a 24 h baseline, starting at lights-off (12 h night and 12 h day), followed by a 12 h dark phase during which the room temperature was lowered from 24 to 4 °C. During the remaining 36 h of the recording, the ambient temperature was restored to 24 °C. The temperature of 4 °C was chosen because it induces a strong and largely selective suppression of REM sleep in rats, a species of comparable size [26]. In a pilot study, we observed that tree shrews handled the 4 °C well but did show a modest drop in body temperature, indicating that the manipulation was effective (unpublished data). An overview of the experimental set-up is shown in Figure 1.

### 2.4. Signal and Data Analyses

EEG, EMG and accelerometery signals were recorded with a sampling frequency of 100 Hz. The data were downloaded from the logger and imported to an automated sleep scoring software (Somnivore Pty. Ltd., Parkville, VIC, Australia). This program uses machine learning algorithms to autoscore the entire recording based on inputs from a human scorer that was unaware of the animal’s identity and the time of the recording. All available data channels were used by the program to determine the vigilance states on a 4 sec basis. The program was trained for a minimum of 100 epochs per vigilance state (wakefulness, NREM sleep or REM sleep). Wakefulness was characterized by low-voltage, high-frequency waves with high power in the theta range (5.25–7.0 Hz), high muscle activity and a high accelerometer output. NREM sleep was characterized by high-EEG-amplitude and low-frequency waves, with greatly reduced muscle tone as compared to waking. During REM sleep, the EEG had low-amplitude and high-frequency waves similar to those visible during wakefulness, where the EMG showed a complete absence of muscle tone. The automated scoring software has been validated for various species, including mammals [29]. To validate the program for tree shrews, F-measures were calculated, including precision, specificity and sensitivity for every vigilant state between the auto-scored and manually scored baseline recordings of the two animals [29]. The F-measure can range from 0 to maximal 1, and in our validation, the F-measure was 0.98 ± 0.005 for wakefulness, 0.94 ± 0.04 for NREM sleep and 0.74 ± 0.19 for REM sleep. Throughout the experiment, the core body temperature was sampled every hour. In the two individuals that had a thermistor on the surface of the brain, the cortical temperature was measured with a sampling rate of 100 Hz.

### 2.5. Statistics

The data were analyzed with linear regression (lm) models using the R stats package [30]. When the ANOVA test of these models gave significant results, we computed additional post hoc Tukey tests to look at specific comparisons [31]. To investigate the effect of low ambient temperatures on the core body temperature and cortical temperature, the temperature values were normalized to the average baseline values to account for inter-individual differences. The results were considered statistically significant when the *p* values were smaller than 0.05. The data and text in the figures are expressed as the mean ± SEM.

## 3. Results

### 3.1. Effects of Cold Exposure on Body and Cortical Temperature

**Figure 2:**
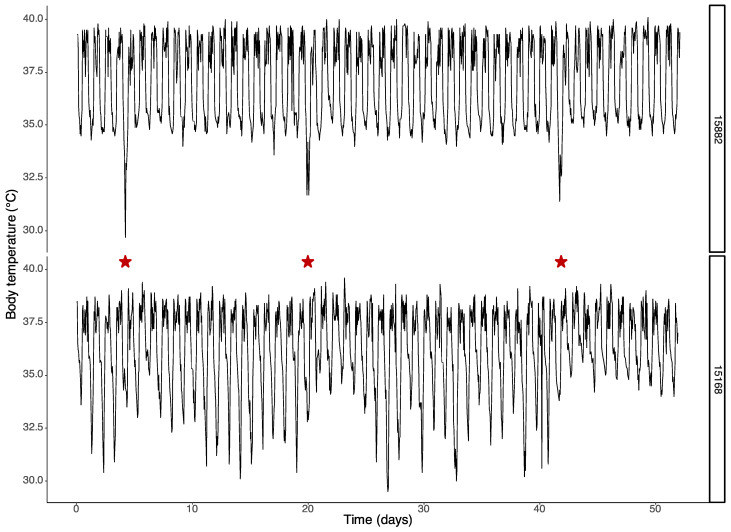
Two examples of daily rhythms in the core body temperature over a period of 50 days. In these recordings, the animals underwent three nightly cold exposures on days 5, 20 and 42 (indicated by a red star). The upper panel shows data from a tree shrew with stable daily rhythms in body temperature, where cold exposure resulted in a steep drop in temperature. The lower panel displays data from another tree shrew that had larger variability in daily core body temperature, and cold exposure resulted in an increase in core body temperature that lasted for several days up to a week.

**Figure 3:**
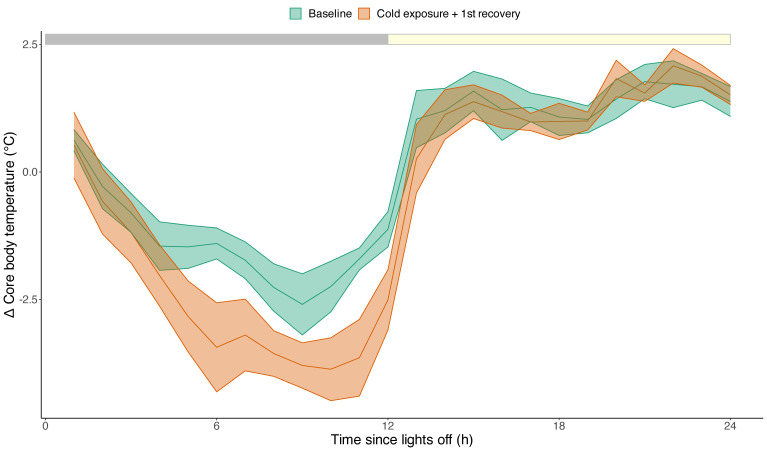
Average change in hourly relative core body temperature in response to cold exposure (n = 6). The red line denotes the baseline condition with ambient temperatures of 24 °C. The blue line denotes nightly cold exposure and the first 12 h of recovery during the light phase, where the ambient temperature was restored to 24 °C. The average response in core body temperature to cold exposure resulted in a total decrease of 1.14 ± 0.7 °C compared to the baseline, especially during the second half of the night, which resulted in a reduction of 1.5 ± 0.8 °C (lm model, *p* = 0.03). Data are plotted as the average ± SEM (shaded areas around the lines). The grey and yellow bar on top reflects the 12:12 light-dark cycle.

Figure 2 shows examples of long-term body temperature recordings in two tree shrews, each subjected to cold exposure on three different nights (indicated by a red star). During cold exposure nights, most tree shrews showed a decrease in body temperature (such as the one in Figure 2, upper panel), but one individual displayed an increase in body temperature (Figure 2, lower panel). The average response of the core body temperature to cold exposure was a decrease of 1.1 ± 0.7 °C compared to the baseline. This was most pronounced in the second half of the night, when the reduction was, on average, 1.5 ± 0.8 °C (lm model, *p* = 0.03; Figure 3).

**Figure 4:**
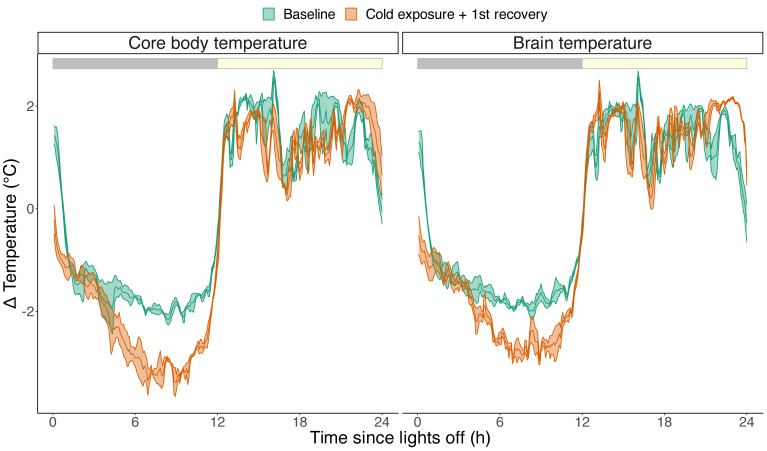
Average change in relative core body temperature (**left**) and brain temperature (**right**) in response to cold exposure sampled per 5 min (*n* = 2). The red line denotes the baseline condition with ambient temperatures of 24 °C. The blue line denotes nightly cold exposure and the first 12 h of recovery during the light phase, where ambient temperature was restored to 24 °C. Cold exposure resulted in an average reduction of 0.9 ± 0.19 °C during the last 6 h of the cold exposure night compared to the baseline for both temperature correlates (lm model, *p* < 0.001). Data are plotted as the average ± SEM (shaded areas around the lines). The grey and yellow bar on top reflects the 12:12 light-dark cycle.

For two individuals, we sampled the body temperature and cortical temperature at a higher frequency of once every 5 min (Figure 4). Similar to the body temperature, the brain temperature also significantly decreased in response to cold exposure (0.74 ± 0.2 °C during the second half of the night; lm model, *p* = 0.037; Figure 4).

### 3.2. Effects of Cold Exposure on Sleep Architecture

**Figure 5:**
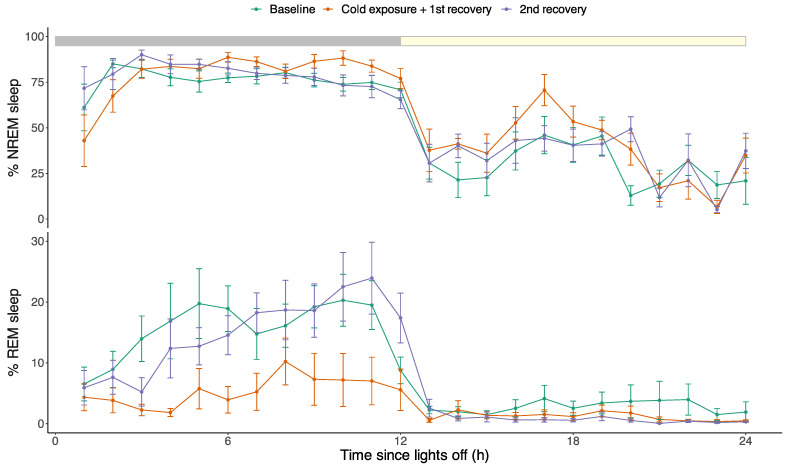
Effects of cold exposure on sleep–wake patterns in tree shrews: (**upper panel**) amount and distribution of NREM sleep; (**lower panel**) amount and distribution of REM sleep. NREM sleep time was unaffected by the cold exposure (lm model, treatment: *p* = 0.48). REM sleep was suppressed from 15.3 ± 4.2% during the baseline night to 5.4 ± 3.0% during the cold night exposure (post hoc test after lm model, *p* < 0.001), without a rebound in REM sleep time during the recovery period (lm model, *p* = 0.65). The grey and yellow bar on top reflects the 12:12 light-dark cycle.

Under baseline conditions, the tree shrews spent most of the night sleeping (Figure 5: 76.1 ± 5.4% NREM sleep, 15.3 ± 4.2% REM sleep, 8.6 ± 5.3% awake). During daytime, the animals were awake for a large proportion of the time but still spent a significant amount of time asleep as well (Figure 5: 29.0 ± 9.7% NREM sleep, 2.8 ± 1.7% REM sleep, 68.2 ± 10.4% awake). As expected, exposure to a low ambient temperature of 4 °C resulted in a highly significant reduction in REM sleep from 15.3 ± 4.2% during the baseline night to 5.4 ± 3.0% during the cold night, which equals a suppression of 64.9% of nighttime REM sleep (post hoc test after lm model, *p* < 0.001). The suppression of REM sleep during the cold night largely translated to a higher amount of wakefulness, which increased from 8.6 ± 5.3% during baseline to 15.5 ± 7.8% during the cold exposure night, i.e., an increase of 79.6% of nighttime wakefulness (post hoc test after lm model, *p* = 0.02). Changes in NREM sleep during the cold exposure night were not significant (lm model, treatment: *p* = 0.48). During the subsequent recovery period when the ambient temperature was returned to 24 °C, there was no rebound in the amount of REM sleep (Figure 5; lm model, *p* = 0.65).

The cold-induced suppression of REM sleep was not dependent on the drop in body temperature (lm model, *p* = 0.87). In fact, even the one animal that displayed an increase in body temperature during the cold night (Figure 2, bottom panel) showed a significant decrease in REM sleep of 90.9%.

## 4. Discussion

This study in the day-active tree shrew aimed to expand the current knowledge on REM sleep regulation by investigating whether selective REM sleep deprivation in this species is followed by a rebound in REM sleep time. Exposure to low ambient temperature resulted in a strong and selective suppression of REM sleep. However, contrary to the expectation, the animals did not show a rebound in REM sleep following cold exposure.

Under baseline conditions, the tree shrews in our study showed a clear daily rhythm in core body temperature, with a large amplitude of about 4~5 °C, in line with what was reported before [18]. The two animals that were equipped with thermistors for recording brain temperature showed a cortical temperature rhythm with an amplitude of a similar magnitude. Additionally, the baseline sleep–wake patterns we found in the current study are in agreement with earlier reports [18,24]. The tree shrews spent over 91% of the dark phase asleep, of which 76% was NREM sleep and 15% was REM sleep. During the light phase, the tree shrews were awake and active for a large part of the time but still spent close to one-third of the time sleeping (mostly NREM sleep).

Cold exposure resulted in a largely selective suppression of REM sleep, while the overall amount of NREM sleep was not affected. These findings are in line with the literature on other mammalian species, showing that cold exposure primarily affects REM sleep. Studies in cats and rats have shown a strong suppression of REM sleep during cold exposure but small or no effect on NREM sleep time [25,26,27,28]. In rats, the NREM sleep EEG deltapower was slightly suppressed during cold exposure [26] and slightly increased during subsequent recovery sleep [26,32].

In the current study, the suppression of REM sleep was not dependent on the drop in body temperature. One animal even had an increase in temperature during the cold exposure night, and consistently so during repeated cold exposures, but still displayed a suppression of REM sleep. It is unknown what caused the extreme variation in the body temperature response to cold exposure.

Importantly, the cold-induced suppression of REM sleep in tree shrews was not followed by a rebound of REM sleep time. This appears to be in agreement with studies on fur seals that have 80 min of REM sleep on land but little or no REM sleep when they are in the water, which can be considered a cold exposure. When they return to land, they show no clear rebound [33]. However, the lack of REM sleep rebound in cold-exposed tree-shrews is in contrast to studies in particular rats that showed a strong REM sleep rebound after cold exposure [25,26,27,33].

It has been suggested that a clear and immediate REM sleep rebound may only occur if the preceding loss of REM sleep reaches a certain threshold [27,28,34]. In the rat, this threshold was found to correspond to a loss of more than 20% of the normal daily amount of REM sleep [27]. Given that our tree shrews lost over 64.9% of their REM sleep during the cold exposure night, an REM sleep rebound was to be expected.

One might wonder whether the tree shrews in our study had a delayed rebound of REM sleep that went unnoticed because we only recorded 36 h of recovery sleep, based on the view that there may be REM sleep homeostasis on a longer time scale [35]. However, studies in rats show both short-term versus long-term REM sleep homeostasis, and with strong REM sleep suppression, the short-term regulation dominates, and there is an immediate rebound that correlates with the loss of REM sleep [25,26,27]. Particularly, in rats exposed to low ambient temperatures that resulted in strong REM sleep suppression, there was a fast compensatory rebound of REM sleep during the first day of recovery that made up for the bulk of REM sleep that was lost and a slower rebound during the following 3 days to fully restore all the remaining REM sleep that was lost. Clearly, our tree shrews had a strong suppression of REM sleep but did not display the immediate rebound that one would expect on the basis of the studies in rats.

It has been suggested that the threshold for an REM sleep rebound, and particularly the fast rebound, may vary between species. Specifically, it was suggested that the threshold correlates with body weight or brain size, with a lower threshold and stronger rebound in smaller animals [27]. However, given that our tree shrews are about the same size as rats, this does not seem to explain the lack of rebound in the current study.

It is important to note that after total sleep deprivation for 6 and 12 h, tree shrews only showed a rebound of NREM sleep, not REM sleep [18]. Similar to the current study, manual sleep deprivation resulted in a substantial loss of REM sleep, which was not recovered. Together, these findings do not support the idea that REM sleep in tree shrews is homeostatically regulated, neither in relation to preceding wakefulness nor in relation preceding NREM sleep. If an REM sleep pressure would build up during waking, one would expect an REM sleep rebound after total sleep deprivation, i.e., prolonged wakefulness. If an REM sleep pressure would build up during NREM sleep, one would expect an REM sleep rebound after selective REM sleep deprivation, i.e., after cold exposure that leaves NREM sleep largely intact. Yet, neither was the case.

The regulation and homeostasis of REM sleep have been questioned on the basis of a wide variety of studies showing that REM sleep may be increased in response to stimuli and conditions that are not associated with a major loss of REM sleep—for example, exposure to dark pulses during the normal light phase [36,37], stress [38,39] and learning tasks [40,41]. The tree shrews may be an interesting model for follow-up studies on REM sleep regulation in relation to such specific stimuli and conditions.

## 5. Conclusions

In conclusion, the current study in a diurnal mammal confirms that the expression of REM sleep is highly sensitive to ambient temperature and also shows that cold exposure can be used as a method to selectively suppress REM sleep. The lack of a recovery response in REM sleep after cold exposure in the tree shrew challenges current theories on REM sleep regulation and does not support the view that REM sleep is homeostatically regulated in this species.

## Data Availability

Data are available upon request.
